# The New and the Old and the New Departure by an Old One

**Published:** 1879-12

**Authors:** J. A. Robinson


THE DENTAL REGISTER.

VOL. XXXIII.] DECEMBER, 1879. NO. 12.

### THE NEW AND THE OLD, AND THE NEW DEPARTURE BY AN OLD ONE.

*Read before the Dental Class of the University of Michigan, November 18, 1879.*

BY J. A. ROBINSON, D. D. S.

"*Prove all things: hold fast that which is good.*"

For the past two years the dental profession has been exercised and somewhat divided as to the best means of preserving the teeth. The best operators have at times been dissatisfied with their best efforts, while less skilful persons have been seeking for something to take the place of gold, and something that was less expensive, and that could be used with less skill and labor. The fault of all this lies somewhat in the persons themselves; perhaps more in the men than in the manner in which the work is performed. The old plan of forty years ago was to carefully prepare the cavities of such teeth as were considered worth saving; extract all doubtful cases; do nothing that would not be sure to keep good the reputation of the dentist; cut the cavities as nearly straight as possible, avoiding all under-cuts, and fill with pellets. The pellets were made, as a rule, of Bull's, now Abbey's, gold foil number four. The gold was cut into strips about three or four times as long as it was wide, and crimped together with the thumb and fore-finger as you would crimp a piece of ribbon or tape; it was then doubled in the middle, the loose ends folded in and given a slight roll between the thumb and finger to make it into a cylinder. These were packed round the marginal walls of the cavity with a graver-pointed, wedged-shaped plugger, leaving them a little above the margin. This process was repeated until the cavity was full, and they were then pressed down until it was tight and hemetically sealed. The best plugger I have ever seen for this purpose is in the form of a hatchet-shaped excavator, broken off at the point to make it square, and the sides and square end brought to a cutting edge on an oil stone. Rest the plugger on your thumb nail, when it will not slip it will always carry the gold before it into place and never pass through it. The writer does not know that the plugger described was used by Harwood and Tucker. It was a blunder, discovered by trying to fill a tooth with a broken excavator. This plugger is to be sharpened as often as it is dull by coming in contact with the tooth or enamel, and it will never fail to carry the gold to the desired place. If the hand is too moist to crimp the gold, do it in a napkin. This may be said to be the Harwood and Tucker plan of forty years ago, and it is substantially the same as practiced in and around Boston to-day.

It may be interesting to some younger men in the profession to know something of Dr. Joshua Tucker, who was the most successful operator upon the old plan, and was, perhaps, the most successful dentist in Boston for more than forty-three years.

Dr. Tucker was born in Winchendom, Mass., in the year 1800. In boyhood he became a fine penman, and 1827 went to Charleston, S. C., to teach writing. His hand was so perfectly trained to do his bidding, that many specimens were taken for copper plate engravings. His first instructions in dentistry was received in Charleston, S. C., from Dr. D. C. Ambler, and he started in practice in that city. At that time there was in Charleston a Dr. Brewster filling teeth in a superior manner, who had learned to pack gold of Dr. Hudson, of Baltimore, and it is perhaps due to Dr. Hudson to say that with him the pellet system began.

Dr. Tucker became so dissatisfied with his own operations when compared with Dr. Brewster's, that he gave up his office and went with Dr. Brewster, and remained one year longer with him and, gave him five hundred dollars for instruction, and in 1829 left Charleston for Cuba to practice his profession.

He returned from Cuba in 1833 on account of cholera, and was invited to a partnership with Dr. Harwood, who was at that time doing a good business in Boston. Harwood and Tucker became the standard for New England, and perhaps for the world, and their office in Hambleton Place was the headquarters for dentistry, as much as Harvard University for education and culture. Harwood and Tucker were the first manufacturers of teeth made of silex and feldspar in America. Tucker shortly left Bos on and graduated at Geneva Medical College, and returned to Boston and joined the Massachusetts Medical Society, and was widely known both in Europe and America. The writer learned to pack gold on the Tucker plan in 1836. and that was the standard in Boston and Lowell, New Bedford and Salem; and whereever Dr. Tucker's fillings were seen, they were identified from all others, as readily as a piece of silk would be from a piece of cotton cloth.

Dr. Tucker adhered to some rules in practice that would hardly be admissible at the present day. He was the great tooth extractor. His advice to the writer as early as 1839 or 1840 was: "Do not try to save teeth that have exposed nerves. You can not afford it. Your reputation must be preserved; what is best for your reputation is best for your patients. If you can save twenty-six, or twenty-four, or even twenty teeth you have done well; extract all doubtful cases, for dead men tell no tales."

I have in my possession at this time the first tooth I ever filled, or tried to fill, in 1836, filled in this manner, and it has never been refilled. It was extracted some three years ago because it became loose and was no longer serviceable. The filling is solid and hard, and there is no appearance of decay or discoloration along the margin of the cavity. I make this statement because some writers of the present day contend there are no fillings that are permanent. That is true, for life itself is only temporary. Dr. Tucker's heart, head and hand were alike skilfully trained to his profession. His singleness of purpose, his careful and delicate manipulation, combined with untiring industry, made him the champion of the old method of saving teeth.

Dr. Tucker was not like the great Atkinson, broad enough to elevate a whole profession all over the land, and with inspiration and enthusiasm enough to stimulate the best minds to do what he is hardly able to accomplish himself. But he was not behind his compeers in thoughts as to the causes of decay, or the manner or formation of tissue or tooth bone. Whenever he advanced an idea, it was always with a modest "I have observed," and his theory that in some cases caries began in the internal structure from deficient calcification, although so stoutly denied by Dr. Arthur, are well worthy of attention and consideration by all enquirers after truth.

He was more of the Reubens type of artist than the Michael Angelo; his forte lay in fine manipulation, great care and patience, combined with gentleness and untiring industry, rather than in great genius.

If we confine ourselves strictly to the old plan, as taught by Harwood and Tucker, we do not necessarily discard the rubber dam, or the engine, or the mallet, or any of the modern appliances for doing the work. Although Dr. Tucker does not think them necessary as in using cohesive foil. The old method consists of using exclusively non-adhesive or soft foil, and it is the packing of the gold and the pellet system. Neither is the instrument for doing the work a graver-pointed plugger. The advantage of a graver-pointed plugger is two-fold: it carries the gold successfully to place, and at the same time leaves the cavity square at the bottom, so that the last pellet will not be likely to come out and thus loosen the whole filling.

Dr. Sage, of Paris, Dr. Foster, of New York, and Dr. Moffet are good representatives of the old method. A heavier instrument is necessary with the old plan than those usually found at the dental depots, as it is done by hand pressure, and the instrument should fit well the hand. All the graduates from Hamilton Place in Boston, have been representatives of the old method, have had a successful practice, and sustained an enviable reputation among the profession. Some one may say that what I have said about Dr. Joshua Tucker had better been reserved till he had passed away. Dr. Tucker has passed away as a dentist. He become entirely blind nearly three years ago, and by having an operation upon his eyes he is able to walk about and read a little in the newspapers. His enthusiasm is as great as ever when talking about his life's work in dentistry, and the fundamental principles of packing gold to save the teeth, and also of extraction. He always winds up his talks with a sigh, saying, "It is all over now." It is sometimes a pleasure to a good man and a faithful worker to know that he is appreciated while he is alive.

I wish I had the ability to state the reasons as Dr. Tucker does for believing that teeth sometimes begin to decay from within, but I have not, so I offer some suggestions of my own in defence of his theory for consideration.

If the development theory be true, there must have been a time when there were no teeth in animal life, or very imperfect representations of human teeth as we find them today. Appearances are the things from which the mind forms an understanding of the forms and functions of life and health, or of disease and decay, and they can not be shaken off but by investigation, and whenever the cause is deep seated, we are often led away into uncertainity and error.

Everything in Nature, by virtue of its origin, must be the recipient of the highest and most perfect life it can attain in health, hence it is only when obstructed by some unnatural cause that it ceases to perform the function allotted to it.

Everything in Nature possesses two inherent qualities, attraction and force. Attraction is the desire to fulfill some agreeable function, and force or life is the power by which that desire is fulfilled. All forms are of the life principle, and when unobstructed they receive the most perfect life that can be attained. The life principle is a spiritual principle; the physical is the form taken to express the life that is useful and tangible. All forms are of the Divine form, and all life is of the Divine life, and when flowing into ultimates, of the conditions are favorable, the highest and most perfect forms are produced. The nails, the hair, and the teeth are the ultimates in man's creation, because they are nearest the mineral, and it is only when insufficient papulum is given out in passing to the ultimate that disease and decay takes place. In the tooth it is when imperfect calcification is carried forward through the infusion of lime salts. There is a time when there is not even the rudiments of the six year old molars, or the second dentition visible in the child, but the thought of the tooth must have been in the mind of the Creator as the ultimate circumference when conception took place, of which he is the center. If this proposition is plain, or it is proven as nearly as any in life can be proven, then it follows that it may be possible that any obstruction to perfect calcification or infusion of the white blood that passes through the tubuli may be so enfeebled that decay may sometimes commence from within, as suggested by Dr. Tucker.

Every careful examiner has observed a bluish tint that is seen through the enamel of the tooth, sometimes extending full one-third across the crown, that is easily broken away with a chisel, when there seems to be no possibility of an opening through a fissure or otherwise, that could cause a decay from without, and hence the reasonableness that teeth sometimes begin to decay from within from imperfect calcification. These thoughts are suggested by the writer in defence of the Tucker theory, and which he could no doubt so much better defend himself if he was present. Dr Tucker's theory of extraction may have been based on the same principle of the farmer, who pulls up a part of the stalks of corn when there are tōo many in a hill, because he had observed that he had a larger number of good ears at his gathering when there are three stalks in a hill than when there are six; that the food being insufficient could not pro- duce both the blade and the fruit. If it is so in vegetable and animal life, why may it not be so in spiritual or intel- lectual? The universe is a form productive of use, and use is the fruit both of vegetable, animal and spiritual life; the highest use is when we have the largest growth of perfect fruit.

Since the agitation of the new departure theory, I have taken a good deal of pains to examine fillings of my own for the past forty years, and to compare the average duration of those filled with gold to those filled with amalgam and other plastic fillings, in order to arrive at the truth of what the advocates of the new departure claim, and am fully per- suaded that those persons who are so enthusiastic on that subject ought to pause and say, "lead us not into tempta- tion," and ask themselves if they can undo what they have done as easily as they have committed a fault, if it is a fault, and try and make amends to a profession that they have honored, and one that has honored them, before stating so confidentially that they are correct, and that the new de- parture is an improvement and a blessing to the world.

It is customary in the medical profession to give personal experience of cases that come under their own observation and it may be pardonable to give a record from my day book and from recent investigation, and a personal examination of the mouths of more than fifty different persons who have been under my care from ten to twenty years, and to give testimony that I doubt not could be corroborated by many dentists if they would take the trouble to hunt up their life's work. The whole country is full of facts in regard to the permanency of gold fillings done by competent operators if they were published, and in making comparisons I do not wish to underrate cement, or to say that it has not at times been a blessing, but to talk of it as an article of exclusive and permanent usefulness to a profession that is respected for useful and ornamental labor among all classes and in all cases, and to say that it is superior to all others seems to me to be absurd. It would seem as though we could get nearer to the truth by statistics of work done with different persons in the same family than in any other way, because their constitutions are more uniform.

A German family with a large number of girls, came under my care not far from ten years ago; the oldest daughter, a girl some sixteen years old, had the two superior central incisors filled with gold. It was a painful operation, and as the mouth was in a bad condition, she gave up the idea of having the work completed. After a little, she went to another dentist in good repute, and had most of her teeth filled with amalgam. She left for Germany to complete her education, and was absent more than two years; when she returned, the amalgam fillings had failed to a large extent, and the teeth too far gone to be saved, and they were left to be replaced with artificial teeth; but the central incisors were as perfect as when filled. The second daughter came about six years since, with teeth that would have been extracted by most dentists; she submitted to the operation of having gold fillings to the number of thirty-three, and four were filled with amalgam. She left for Europe, and was absent more than three yeurs; while absent, two gold fillings failed and were replaced abroad with amalgam. On her return home, more than a year since, all the gold fillings were good, and all the teeth with amalgam in a state of decay. The third daughter came about the same time, with teeth so bad that seven were extracted, the balance were filled with gold as far back as the bicuspids on right side, and first molars on left side, and seven filled with amalgam. The amalgam fillings have had to be replaced to save them more than once, while the gold ones are as good, to all appearance, as when first put in. The fourth daughter, a girl fourteen years old, was the next to visit me for dental work, July and August, 1876. Only two teeth had been extracted; they seemed stronger than the others, but had commenced to decay in almost all approximal surfaces, and many fissures. Thirty-one cavities were filled with gold that remain good.

A boy from the same family, about twelve years old, went to another dentist in good standing, and had the first inferior bicuspid and six year old molars filled on approximal cavities, with amalgam, and within one year he returned to me with both fillings loose, and both the teeth aching. The nerves were destroyed and the teeth filled with gold, which remain as bright and perfect as though they were done but yesterday.

The eldest son had work done in gold to the amount of seventy-five dollars, before the daughters, and only one has ever been refilled. Four years since, two young ladies fourteen and sixteen years of age, came for dental operations with their mother, with the request that the front teeth should be filled with gold, all the back teeth filled, as she said, with silver, as the mother had teeth filled with silver many years ago that were good now. I found on examination, teeth in the mouth of the mother filled with silver that looked as though they had been filled a number of years; the proximal cavities were crumbling a little at the margins, and one or two loose. I advised the mother that silver would not hold them, as the girls were delicate and the teeth frail. My arguments were of no avail, and I did the best I could, using Lawrence's amalgam, and two with amalgam made by a Chicago refining company, said to be part gold and platinum. I also filled the crown cavities in both superior and inferior molars with tin foil. The tin foil fillings remain good, the gold ones perfect, and the amalgam fillings did not last quite two years, when they were taken out and replaced with gold that has saved the teeth. In speaking of plastic materials, I include all that I have had experience with; for Weston's insoluble cement, wherever it is exposed to wear, or in approximal cavities, sooner or later washes away. My brother, Dr. B. T. Robinson, has a gold filling in the buccal surface of right superior wisdom tooth inserted in 1846, that has never been re-filled, done with soft foil and it is good now.

While on a visit to Dr. McKellops, in St. Louis, some ten years since, I saw at his office a lady from Phildelphia, who had called for dental operations. He called my attention to her teeth, for they were in a shocking bad condition, and remarked that Dr.— though a professor in a dental college could not successfully fill teeth.

My attention was called to the subject of dentistry by the loss of most of my own teeth before the age of nineteen years. Since marriage we have had eight children to grow up and their ages are between forty-seven and twenty-eight years; of the four daughters, three of whom are married and have large families. not one of them has ever lost a tooth except one or two wisdom teeth; and although they present the appearance of gold mouths, they have never had the tooth ache or had teeth extracted. My two sons who have been absent from home at times from five to ten years have had some few extracted. but my third son who is now thirty-six years has never lost a tooth or had one extracted though the gold fillings are very plenty in all parts of his mouth, and we have never had a child, but has had teeth filled, before they were eight years old.

A gentleman well known in this city had six front incisors built down with gold twenty-one years ago, and four of the six remain pretty good, and he has had no other teeth in the upper jaw for mastication since the work was done. They have been rebuilt down quite a number of times since they were first done. My wife is more than seventy years old, and has never worn an artificial tooth except one lateral incisor, placed upon four gold screws, twelve years ago. There is a good number of contour fillings in this city that are clear and bright, that have been worn from sixteen to twenty years. A gentleman called at my office about four years since, and brought with him in his mouth seventeen gold fillings out of nineteen that I had the honor of filling in 1840; only two had failed, and his teeth had had no fillings since that time. As I learned the old pellet system, I have always packed the walls of the cavities with pellets after the Tucker plan, and finished with cohesive gold, for a good many years, calling it the Eclectic method, scarcely ever making a retaining point, believing that one cavity w[illegible] enough to take care of, and for the past few years filling all proximal cavities against an orange wood wedge, as it never fails to hold the gold in place, and is sufficiently elastic to allow the material to come flush with the margin for the finish.

I read an article a short time since, in which the writer went on to say: "If every dentist would speak his mind and tell the truth, there was not one in the whole land but would say that amalgam was better than gold."

Are the dentists a set of liars and hypocrites? Would it not be just as unfair to assert that there never was a man in favor of amalgam, or that would prefer amalgam, who could successfully pack gold? Do you believe Dr. McKellops, of St. Louis, or Drs. Allport and Cushing, of Chicago, or Dr. Butler, or Dr. J. E. Robinson, or Dr. Jennings, of Cleveland, or Prof. Watling, of Ypsilanti, or Corydon Palmer, of Warren, Ohio, or a hundred others we could mention, who have had the experience of a quarter of a century, are dishonest men? Or that they do not act up to their highest convictions of duty and love for their profession and their patients? The question is not what will sometimes save very poor teeth, but what we shall teach the profession and the world. But the new departure, as it is called, is not a new thing.

There was a Dr. Mann, in Boston, who got all Boston in a blaze for cheap dentistry more than forty years ago with this same theory, and the same amalgam under the name of "Sucsedanium Cement," and it went down because it was not fit to survive; and what is not that, will be ephemeral and pass away in like manner.

I do not believe it will be contended that a person who is a good operator in gold, can not fill teeth well with plastic fillings; but it may be possible that a man may be able to do well with amalgam, who is entirely incompetent to preserve teeth with gold. What we would state is, that after more than forty years' experience, and after many failures when I thought I was doing my best, I find on close examination, among a large number of patrons, that the gold fillings, as a rule, preserve the teeth, both good and bad, hard and soft, frail and strong, more than four times as long, and as well, and as substantially, as any other substance that I am acquainted with.

The dental profession more than most others, needs the education of the imagination; the thoughts are the fruits of our lives, and man's real nature is only his organized affections; and as in the leaves and blossoms of the trees there will be many that will not reach a state of fixedness, and bear perfect fruit. So it will be with our work. We should all labor for the best and highest good to ourselves and the world. There will never be, there can never be, a superabundance of good dentists. "Strive to enter into the straight gate." It is not what we have been able to do, but what we have tried to do; and then we do not always do our best. Ill health, fatigue, distracted thoughts, love of ease, are the great enemies of our profession, and now the great wound has been inflicted in the house of its friend. The embodiment of stupidity has found a mouthpiece in a high place, and as an offset to the assurance of the advocates of the new departure, I do solemnly aver that I do not believe there has ever been one case since dentistry began, but would have been done better, (if worth doing at all) with gold than any other material yet discovered, if done by a competent person. I do not condem these men: they are in the good of life, and will embrace the truth, when their mistakes are made known. I think they will discover that they are far away from the center and that the circumference will be like all amalgam fillings, full of imperfections and failures. We can not be wise without we are good; but we may be good and not very wise. Truth produces order, and error disorder. We must learn to love and guard well our profession, for we can not truly love without an object for the affections; and man is the form of his highest affections. It has not been my aim to speak lightly of any material that can help the world, but to discuss the merits of the two and their relation to each other, and give each its proper place among the blessings to mankind, and to stimulate those who have made failures like myself in all methods, to labor hard to overcome every difficulty in order that they may be accounted worthy to be called honorable members of a most honorable profession. Of all the amalgams that have been made, I do not believe there has ever been a better one than that introduced by Dr. Mann, of Boston, or that is less destructive to the teeth than that made of an old fashioned quarter of a dollar, because there is not the difference in oxidation between the mercury and the silver that exists between some of the materials now used in the popular amalgams.

I am aware that we shall be called "gold bugs" and fossils," and I have not forgotten that a member of Parliament was presented with a service of solid gold for the defence of slavery in the West Indian Islands, and that his children and grand children are to-day ashamed of the donation. It is not the improved implements or methods; but the amalgam itself that I am against. Only two weeks ago I passed through a woolen factory and saw the stalwart spinner walking his six feet backward and forward and tending two hundred spindles with the same kind of bobbins and rolls, to the same music, only intensified by machinery, that grandmother used, when the country was new; and the singing of the carding machine as the twenty-four cards drew out the woolen fibres, was the same in kind only intensified that my mother used over her lap, when we were children. The shuttle was the same only it was driven by machinery instead of the human hand. The scouring was the same only the water was forced upon the wool in immense quantities instead of being pounded out in a wash tub. The coloring was the same and the twisting of the yarn was the same ; but there was one thing that I saw that was not seen before; there lay in one corner of the room an immense pile of stuff that resembled wool that was without fiber, and that could not be spun or woven without it was mixed with clear nice wool.

It was shoddy; it was made out of old worn out cloth and worth very little except to have the apperance of cloth, when it was finished, and to cheat the world by being cheap, and representing a thing and a quality, that it did not possess. In discussing this question I do not take into the account poverty or the inability if some persons to have work done in the best manner, but the intrinsic value of each material to save teeth.

It is better to live in a log house than to live out of doors in the cold of winter; what we want is, a new method of an old system that is true; and gold is the most indistructible of all metals, nothing, but "Aqua Regia" will destroy it, and I hope the time is not far distant, when something will be discovered, that will have an affinity for the tooth, and will restore it its original usefulness, and will come within the reach of all classes. But it has not yet been discovered that I have heard. If there are those, who have not the faculty to do the work or to discern the difference, there is between the two, so much the worse for the men. I leave this subject for your consideration, only hoping that you will be able in your day and generation to excell any of your predecessors, and so move on to the perfection that the times and the subject so well deserve.

